# Competency-based education – the reform of postgraduate medical training in Switzerland

**DOI:** 10.3205/zma001717

**Published:** 2024-11-16

**Authors:** Fabienne Schwitz, Monika Brodmann Maeder, Eva K. Hennel

**Affiliations:** 1 Bern University Hospital, Inselspital, Department of Cardiology, Bern, Switzerland; 2 SIWF Schweizerisches Institut für ärztliche Weiter- und Fortbildung, Bern, Switzerland; 3 University of Bern, Bern, Switzerland; 4 University of Bern, Institute for Medical Education, Department for Assessment and Evaluation, Bern, Switzerland

**Keywords:** postgraduate medical education, CBME, EPA, curriculum, faculty development

## Abstract

**Objective:**

Medical training in Switzerland is currently undergoing change. The postgraduate education curricula of all medical specialties are being converted to competency-based medical education (CBME). Entrustable Professional Activities (EPA) are used to assess competencies. EPAs describe specific professional tasks that are assigned to postgraduate trainees once they have achieved sufficient competencies.

**Methodology and results:**

The article describes how the didactic building blocks are joined to create competency-based teaching and how the implementation takes place.

The project is described using the Kern cycle. The first two steps, problem identification and targeted needs assessment, are presented in the project description section, the other four steps in the results. Concrete details are given using examples from the cardiology curriculum.

**Conclusion:**

The conversion of medical training in Switzerland to competency-based teaching is an important step that is urgently needed but complex. The long-term plan of the Swiss Institute for Postgraduate and Continuing Medical Education (SIWF) consists not only of structural steps but also cultural change. The first two years of the conversion were successful. In collaboration with the specialist societies, postgraduate curricula are being converted to EPA-based learning objectives, the didactic training for postgraduate teaching staff adapted accordingly and feedback from learners is continuously gathered. The implementation process has begun. Additional data will be collected as the project proceeds. Using experience already gained internationally and by specialist societies which have already taken this step as benchmarks is critical for other specialties and training centres that are still to follow.

## Zusammenfassung

**Zielsetzung:**

Die ärztliche Weiterbildung der Schweiz ist derzeit im Wandel: Die Weiterbildungscurricula aller medizinischen Fachgebiete werden auf eine kompetenzbasierte Lehre (Competency-Based Medical Education, CBME) umgestellt. Zur Bewertung der Kompetenzen werden Entrustable Professional Activities (EPA) verwendet. EPAs beschreiben spezifische berufliche Aufgaben, die den Weiterzubildenden nach Erreichen ausreichender Kompetenzen übertragen werden.

**Methodik und Ergebnisse:**

Der Artikel stellt dar, wie die didaktischen Bausteine sich zu einer kompetenzbasierten Lehre zusammenfügen sollen und wie die Implementierung vonstattengeht.

Das Projekt wird anhand des Kern-Zyklus beschrieben. Die ersten beiden Schritte „Problem Identification“ und „Targeted needs Assessment“ werden im Abschnitt der Projektbeschreibung vorgestellt, die weiteren vier Schritte in den Ergebnissen. Konkrete Details beschreiben wir anhand der Beispiele aus dem Curriculum der Kardiologie.

**Schlussfolgerung:**

Die Umstellung der ärztlichen Weiterbildung der Schweiz hin zu einer kompetenzbasierten Lehre ist ein wichtiger Schritt, der dringend notwendig aber komplex ist. Der langfristige Plan des Schweizerischen Instituts für ärztliche Weiter- und Fortbildung (SIWF) umfasst nicht nur strukturelle Schritte, sondern auch einen kulturellen Wandel. Die ersten zwei Jahre der Umstellung verliefen positiv: In Zusammenarbeit mit den Fachgesellschaften werden Weiterbildungscurricula auf EPA-basierte Lernziele umgestellt, die didaktische Schulung der Weiterbildenden entsprechend angepasst und kontinuierlich das Feedback der Lernenden eingeholt. Der Implementierungsprozess hat begonnen. Weitere Daten werden im Verlauf erhoben. Die Orientierung an internationalen Erfahrungen und an den Fachgesellschaften, die den Schritt bereits gewagt haben, bietet eine wichtige Hilfe für die Fachgesellschaften und Weiterbildungsstätten, die noch folgen werden.

## 1. Introduction

Medical training in Switzerland is currently undergoing change. The postgraduate education curricula of all medical specialties are gradually being converted to competency-based medical education (CBME). Competency-based medical education is a didactic approach that focuses on the development and assessment of specific skills and competencies required to successfully practice a profession [1]. In the past, medical training was regulated primarily on the basis of completed terms and the number of examinations or interventions, analogous to the situation in Germany and Austria. For example, the curriculum in cardiology requires trainees to complete two years of basic training in internal medicine and four years of specialist training in cardiology. In addition, at least 500 ECGs had to be performed and assessed [2]. The new competency-based teaching model prioritizes the learners and their competencies instead of the training time required [3].

Entrustable Professional Activities (EPA) are used to assess competencies. EPAs describe specific professional tasks that are assigned to postgraduate trainees after they have acquired sufficient competencies [4]. EPAs should be observable, measurable, completed within a certain time frame and suitable for assessment by teaching staff [5]. A concrete example of an EPA is the observation of a “treatment of a patient with symptoms and signs of cardiac insufficiency”. Postgraduate teaching staff then assess the degree of independence with which residents should be able to complete the EPA. EPAs are intended to provide a safe framework to gradually increase the trainees’ autonomy.

With the CanMEDS framework [6], Canada has done a lot of groundwork for the introduction of competency-based teaching worldwide since the 1990s [7]. The concept of the EPA was developed in the Netherlands and was subsequently integrated into national curricula [8]. CBME and EPAs have been adopted primarily in Anglo-American countries [9], [10], [11].

In Europe, the European Union of Medical Specialists (UEMS) organized its first conference with the aim of improving postgraduate medical education and integrating competency-based teaching and EPAs in 2023 [12]. Together with the Netherlands and Finland, Switzerland is now at the forefront of introducing structured, competency-based medical education in Europe [13].

The SIWF is responsible for implementing this change in Switzerland. The SIWF is an autonomous body of the Swiss Medical Association (FMH) and has a federal mandate as regards postgraduate and continuing medical education. Among other things, the SIWF is revising postgraduate medical training programmes in close cooperation with the specialist societies.

In the literature on CBME and EPAs, in addition to numerous reports on the development or introduction of EPAs, there are only a few publications that deal with complex national implementation strategies [14]. Using the example of the introduction of CBME in Switzerland, we would like to show how individual didactic building blocks can be joined and implemented to produce competency-based medical education.

## 2. Project description

The project is described using the Kern cycle, a six-step approach for curriculum development [15]. The first two steps, problem identification and targeted needs assessment, are presented in this part of the paper, the other four steps in the results section concrete details are described using the example of cardiology.

### 2.1. Problem identification

Since 2018, Switzerland has run competency-based medical education using the PROFILES framework [16], [https://www.profilesmed.ch/], which means that those starting postgraduate medical education today have already experienced competency-based teaching. Postgraduate medical education now must follow lead in order to build on the achievements of undergraduate medical education.

Although it is common practice to tie the completion of postgraduate education to a period of time or a fixed number of interventions, this has little validity [17]. Internationally and in Switzerland, it has been observed that postgraduates are “passed through” but that gaps in skills or knowledge remain. In addition, according to a recent survey by the Association of Swiss Residents and Senior Physicians (VSAO), only around a fifth of those in postgraduate medical education complete the required four hours of structured training per week [18]. As a result, the system fails both the postgraduate trainees and the health care system. For example, postgraduate trainees are often observed as having weaknesses in communication skills [19]. In contrast, by explicitly looking at individual skills, such as communication, gaps are actively tackled. 

There is an increasing desire among postgraduate trainees for a better balance between postgraduate medical education and their private lives. With competency-based education, where the length spent in postgraduate medical education is less central, one would also expect it to be easier to complete training on a part-time basis, with more flexibility and the options for taking time out during postgraduate medical education. 

### 2.2. Targeted needs assessment: Who are the targeted learners and stakeholders?

#### 2.2.1. Target group

The new curriculum affects all physicians who complete their postgraduate medical education in Switzerland. It includes postgraduate training in all specialist areas and all types of recognized postgraduate training centres, i.e. hospitals of various sizes as well as outpatient postgraduate training centres. 

#### 2.2.2. Stakeholders

In addition to postgraduate trainees, the change also affects the specialist societies and postgraduate training centres. 

## 3. Results

### 3.1. Goals and objectives

In the long term, the curricula of all 45 Swiss federal medical specialisations are to be converted. EPAs are already being prepared or used for 28 of the 45 medical specialisations and 5 of the 52 subspecialties. The specialist societies, each accompanied by a member of the EPA commission, are at different stages of this process, ranging from first contact with the commission to EPAs that are already in use. In addition, a competency-based core surgical curriculum has been developed by surgical specialist societies [20].

### 3.2. Educational strategies 

#### 3.2.1. Involvement of the various stakeholders 

Residents, supervisors, postgraduate training centres and specialist societies were repeatedly involved and in a variety of ways. This included formats such as discussions, lectures, videos [https://youtu.be/uaPKgGUzK2Y] and publications on the topic [21], [22], [23], [24], [25], [26], [27], [28], [29], especially in the Swiss Medical Journal, in order to reach a broad target group. The annual MedEd Symposium was also used to offer lectures and workshops on the topic.

#### 3.2.2. EPA commission 

The commission initially consisted of people who were already active in EPAs in Switzerland and later expanded to be able to support a larger number of specialist societies. People who also had experience with EPAs and had a Master of Medical Education were later added to ensure the ability to give well-founded advice.

The commission has achieved a number of goals to date. 28 specialist societies have already been advised on the development of EPAs, a standard grid for EPAs was developed (2019, updated April 2022) [30] and a recommendation for examinations using EPAs was formulated [31]. 

### 3.3. Implementation 

#### 3.3.1. (Political) support

*Federal Office of Public Health (BAG) of the Federal Department of Home Affairs (EDI):* The collaboration with the EDI was constructive and effective from the start. For example, in the OBSAN 2023 report [32] “Future body and demand for specialist doctors in Switzerland”, the EDI recommended that competency-based medical training should be implemented in all specialties and training centres. And as part of the ongoing accreditation of all federal postgraduate medical education programmes by the BAG and the EDI, Standard 12 asked how far the introduction of competency-based teaching had progressed in the individual specialties [33].

*Specialist societies: *An important partner who has to be fully on board are the specialist societies. Regular mandatory accreditation schemes could be used as one of the points of cooperation. All postgraduate medical curricula are accredited every seven years, most recently in 2018. The 2025 accreditation is currently underway. The specialist societies’ self-assessment report describes 12 target standards, one of which concerns the change to competency-based medical education. The long-term goal is to convince all specialist societies of competency-based medical education. The SIWF and the EPA Commission support the specialist societies in their implementation with advice. Some particularly complex pilot projects are also supported financially. 

*International advisory board: *An international advisory board, staffed by people with experience of CBME and EPAs, and the Royal College of Physicians of London are supporting the process. Further support is provided by the Accreditation Council for Continuing Medical Education (ACCME), the ICBME Collaborators (International Competency-Based Medical Education) and the European Union of Medical Specialists (Union Européenne des Médecins Spécialistes, UEMS).

*Change agents:* The project followed in the wake of the introduction of CBME into medical education. The people who were therefore primarily involved as change agents were those who had already gained experience with CBME in university education, i.e. in the creation and implementation of the PROFILES framework. 

*Postgraduate trainees: *The perspectives of postgraduate trainees was specifically included, represented by the Association of Swiss Residents and Senior Physicians (VSAO), which supports the project.

#### 3.3.2. Identification and procurement of resources

Faculty development for postgraduate teaching staff as a resource: Workshops for postgraduate teaching staff have been held with the Royal College of Physicians of London since 2012, supplemented by a Swiss team since 2018. By 2023, management was handed over to a Swiss team, the structure of the courses was changed and the team was expanded. Additional course locations were added, and in addition to English as the medium of instruction, German, French and Italian are now offered too. The following are taught, among other things: Clinical teaching, assessment and feedback, leadership and learning in virtual spaces [34].

#### 3.3.3. Identification and overcoming obstacles 

Instead of making the use of EPAs compulsory, this first phase of implementation is based on voluntary participation. We hope that courageous and committed specialist societies and hospitals will act as trailblazers who will convince others to follow suit. Competency-based medical education will only be made compulsory when around 80% of specialist societies and sites are participating.

#### 3.3.4. Introduction of the curriculum and piloting (using cardiology as an example)

The specialist society for Cardiology is the first in Switzerland to include and implement EPAs in its postgraduate medical education programme. In this example, we take a closer look at the creation and introduction of the EPAs. 

**Figure 1:**
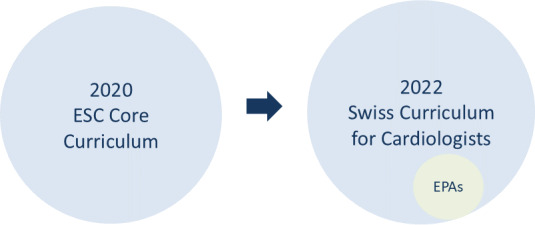
Swiss curriculum for cardiologists Note: Development of the Swiss postgraduate curriculum for cardiology based on the European curriculum. EPAs are an integral part of the Swiss curriculum.

The Swiss Curriculum for Cardiologists [2] was based on the competency-based 2020 European core curriculum for the cardiologist [35], see figure 1 (Fig. 1). It is structured according to the principles of competencies and contains 62 EPAs which must be mastered at different levels of independence. Table 1 (Tab. 1) shows an excerpt from the cardiology postgraduate curriculum. 

**Figure 2:**
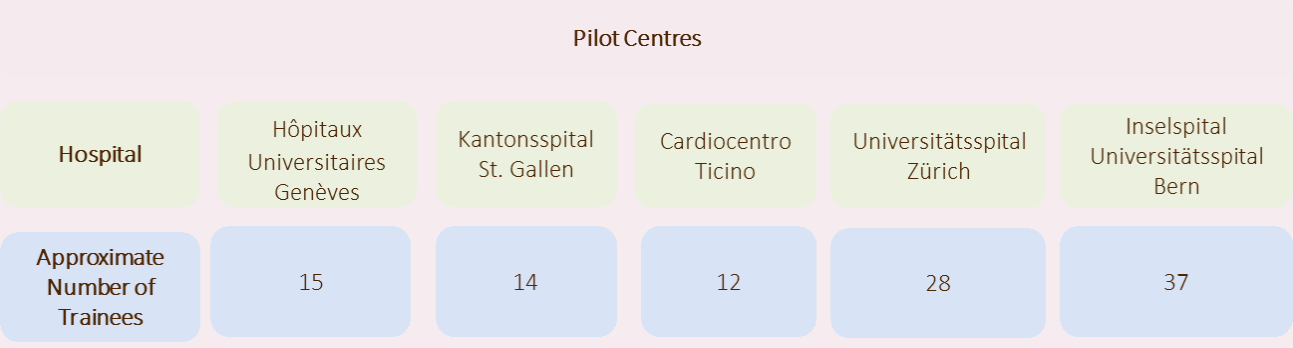
Pilot centres for the introduction of EPAs in cardiology

The introduction of EPAs in cardiology began in 2022 at five pilot centres in different regions of Switzerland, see figure 2 (Fig. 2). All pilot centres are certified training centres for cardiology in category A, i.e. cardiology clinics of university hospitals or comparable centres.

#### 3.3.5. Administrative measures

So far, no additional administrative measures have been necessary. 

#### 3.3.6. Optimization through successive sub-steps

The implementation of the EPAs has already been planned with several sub-steps to facilitate the transition. The previous intervention lists and the associated figures on how often each intervention must be carried out have not yet been completely replaced by EPAs. For the time being, a mixed form is being used that includes both the EPAs with levels and the previous indication of a guideline number for the implementation of the interventions. These numbers can be omitted but only at a much later stage.

### 3.4. Evaluation and feedback

According to the Kern cycle, evaluation means both the individual assessment of the learners and the evaluation of the curriculum (programme evaluation). The evaluation planned by the SIWF also includes both and follows the specific framework developed by the ICBME collaborators [36]. This consists of the following five pillars: Outcome competencies, sequenced progression, tailored learning experiences, competency-focused instruction and programmatic assessment. Not all pillars can currently be examined. We report by way of example which data is obtained by using the EPA app.

**Figure 3:**
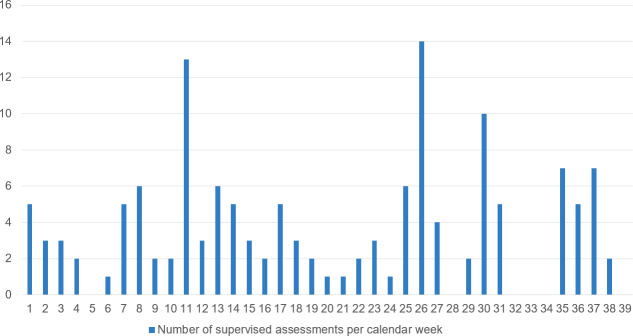
Use of the EPAs Note: Number of supervised assessments using the app per calendar week. Data from a pilot location is shown for the years 2022/2023.

The number of active postgraduate trainees and supervisors was recorded monthly via the application used to carry out and document the EPAs (preparedEPA) [https://www.prepared.app/epa], see table 2 (Tab. 2). Since January 2023 an overview page has been available in the app to those responsible for postgraduate training in the pilot centres. This allows them to see which postgraduate trainees and postgraduate teaching staff are registered and how many assessments were carried out per calendar week (see figure 3 (Fig. 3)) and per person being trained in their clinic. This overview page offers the opportunity to monitor the use of the app and, if necessary, to support both individual postgraduate trainees and postgraduate teaching staff at the training centres.

For further evaluation, the annual survey of postgraduate trainees will be used, which has a very good response rate of around 70% [37]. The next survey will take place in summer 2024. We expect the results in December 2024. The targeted survey of new postgraduate trainees and postgraduate teaching staff at selected training centres before and after the implementation of EPAs will also provide important data. The survey prior the introduction of the EPAs has already taken place in some specialisations and postgraduate training centres or is currently underway. The collection of post-data depends on the progress at the locations and is expected to take place in autumn 2024.

## 4, Discussion

### 4.1. Reflection on the process to date 

Although the plan envisaged a lengthy process, many of the training centres and specialist societies were won over by the competency-based teaching model more quickly than expected and decided to switch early on. In some specialist societies, however, implementation was significantly slower than expected. The speed with which EPAs are created and implemented in individual specialist societies depends on a combination of factors such as technical complexity, available resources, organizational structure, cultural acceptance, existing educational structures and regulatory requirements. Individual support for the specialist societies will be necessary and will be guaranteed by the EPA Commission, among others. Neither the postgraduate training centres nor the specialist societies can make the switch to competency-based teaching alone. In particular with regard to the implementation of EPAs in everyday clinical practice, close cooperation between the stakeholders is required. Postgraduate trainees must be prepared for this and postgraduate teaching staff those carrying out further training must be trained accordingly.

### 4.2. What are the consequences of the change for those affected?

Postgraduate trainees fall into different categories: Graduates who are just starting their postgraduate medical education will already be familiar with competency-based teaching and are therefore continuing in the same vein. They bring the knowledge and commitment to making competency-based teaching permanent and are therefore important change agents. Postgraduate trainees who have already commenced their training may not always find the changing process beneficial. They began their postgraduate training under the old curriculum and the change to the new system may be perceived as additional work. For this group of postgraduate trainees, it is essential that there is a clear and practical transitional provision.

Using the cardiology pilot centres as an example, it shows that the implementation process must be monitored over a longer period of time. Regular information events for those commencing postgraduate training and postgraduate teaching staff in the individual training centres, as well as an active exchange between those responsible for postgraduate training in the various clinics made the EPAs more tangible and spread information about the use of the instruments. At the beginning of the introduction of the EPAs in cardiology, for example, the possibility of self-assessment was hardly known (see table 2 (Tab. 2)). A year later, it was clear that the self-assessment instrument was being used more frequently than at the beginning. 

Feedback from postgraduate trainees is necessary, in the first instance to take their needs into account and secondly to optimize the process. It is therefore particularly important to us to keep an eye on this group and to accompany their progression on an ongoing basis. 

In the future, postgraduate teaching staff should be even better prepared for their role, including the competent use of the competency-based elements of the teaching. The train-the-trainer courses will be further expanded.

The training centres in the in-patient sector accredited by the SIWF are responsible for providing postgraduate medical training. In recent years, there has been a move to professionalization in the field of medical education, with offers ranging from one-day courses to certificates of advanced studies and master’s degree programmes. In future, postgraduate education centres will be required to train at least one person in a managerial position in medical education as part of the accreditation process. 

From 2022, postgraduate medical education in in-patient training centres will benefit as the financing of training by the individual cantons is now regulated at the national level. The cantons are required to provide at least CHF 15,000 per doctor per year in postgraduate medical education for the provision of structured postgraduate training [38]. The SIWF has since also specified what is considered structured postgraduate training from a didactic point of view [39].

The specialist societies are faced with the challenge of having to adapt the curricula. By now, it is now possible to use the experience of other specialist societies and international examples as guidance. The SIWF readily provides advice in order to jointly revise the postgraduate programmes. The EPA Commission can be contacted any time at EPA@siwf.ch.

### 4.3. Looking to the future

The visible aspect of the introduction of competency-based teaching is complex structural change. Among other things, the conversion of postgraduate medical education, the adaptation of processes at the postgraduate training centres and the expansion of the Train the Trainer courses are necessary. This can be visualized by looking at fixed milestones and goals. The second process, which takes place simultaneously, is more difficult to capture. We try to document this as far as possible through Switzerland-wide surveys and through quantitative and qualitative data. What we mean by this is the necessary cultural change. The understanding of teaching does not change quickly and a long-term strategy is required to shape the transition and long-term observation to capture this change. The SIWF’s plans for redesigning teaching are currently poised to run for approximately 10 years in order to be able to fully accompany the process and continuously evaluate it.

The second look into the future concerns the embedding of our postgraduate medical education internationally. There is already a substantial migration of doctors and this will continue to affect us in the future. The competency-based nature of postgraduate medical education can be a great help in the long term when it comes to making medical practice comparable internationally. For the time being, however, the changeover will raise questions as long as specialisations are awarded according to different criteria, i.e. time-based versus competency-based.

## 5. Conclusion

The conversion of medical training in Switzerland to competency-based teaching is an important step that is urgently needed but complex. The long-term plan of the Swiss Institute for Postgraduate Education and Workplace Training (SIWF) consists not only of structural steps but also cultural change. Among other things, in cooperation with the specialist societies, all postgraduate medical education curricula are being converted to EPA-based learning objectives, the didactic training of postgraduate teaching staff is being adapted accordingly and feedback from learners is being obtained on an ongoing basis. The first two years of the conversion were successful. The first measures towards competency-based teaching were implemented. EPAs were introduced as an important tool, although the process of leaving behind “graduation by numbers” (number of examinations or interventions required to complete specialist training) is not fully complete as yet. We assume that at a later stage, once the switch to competencies has been completed, graduation by numbers will be eliminated altogether. Further data will be collected as the process progresses. Using experience already gained internationally and by specialist societies which have already taken this step as benchmarks is critical for specialist societies and postgraduate education institutions that are still to follow.

## Authors’ ORCIDs

- Fabienne Schwitz: [0000-0001-6802-0700]
- Monika Brodmann Maeder: [0000-0001-5608-7887]
- Eva K. Hennel: [0000-0002-7625-5785]

## Competing interests

The authors declare that they have no competing interests. 

## Figures and Tables

**Table 1:**
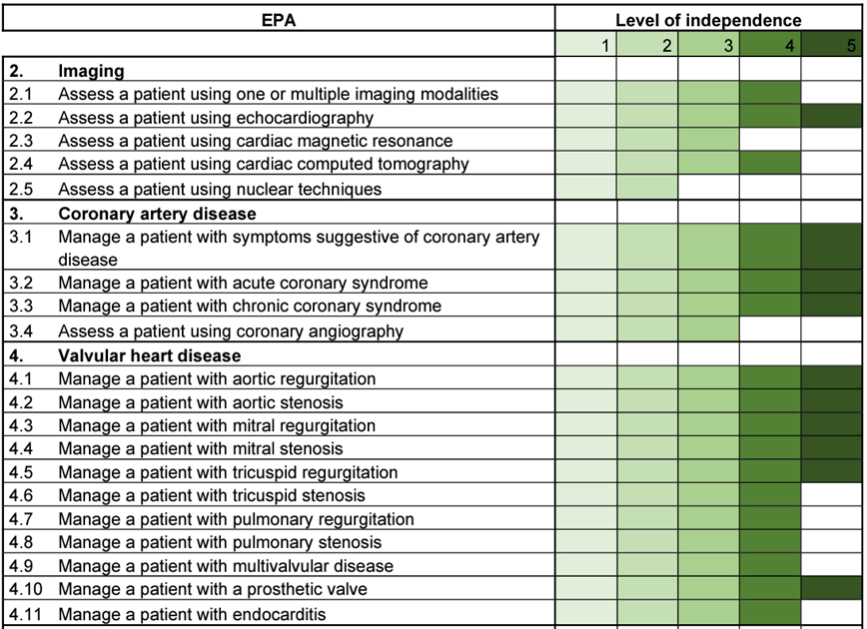
Extract from the postgraduate curriculum for cardiology [https://www.siwf.ch/files/pdf19/kardiologie_version_internet_d.pdf] Note: Depending on the EPA, different levels of independence are aimed for. Reading example: For EPA 3.4 “Assess a patient using coronary angiography”, level of independence 3 is required. Postgraduate trainees do not have to do this completely independently but under indirect supervision. This is different, for example, for EPA 4.2 “Manage a patient with aortic stenosis”. Here, level of independence 5 is the target. This activity must be carried out independently without supervision and they must also be able to take on the supervisory role.

**Table 2:**
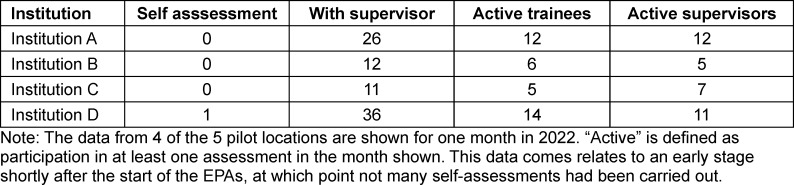
Implementation of EPAs per location
